# Pathogenicity and virulence of the liver flukes *Fasciola hepatica* and *Fasciola* *Gigantica* that cause the zoonosis Fasciolosis

**DOI:** 10.1080/21505594.2021.1996520

**Published:** 2021-11-22

**Authors:** Richard Lalor, Krystyna Cwiklinski, Nichola Eliza Davies Calvani, Amber Dorey, Siobhán Hamon, Jesús López Corrales, John Pius Dalton, Carolina De Marco Verissimo

**Affiliations:** Molecular Parasitology Laboratory, Centre for One Health and Ryan Institute, National University of Ireland Galway, Galway, Ireland

**Keywords:** Parasites, fasciola hepatica, fasciola gigantica, liver fluke, host-parasite interplay

## Abstract

Fasciolosis caused by the liver flukes *Fasciola hepatica* and *Fasciola gigantica* is one of the most important neglected parasitic diseases of humans and animals. The ability of the parasites to infect and multiply in their intermediate snail hosts, and their adaptation to a wide variety of mammalian definitive hosts contribute to their high transmissibility and distribution. Within the mammalian host, the trauma caused by the immature flukes burrowing through the liver parenchyma is associated with most of the pathogenesis. Similarly, the feeding activity and the physical presence of large flukes in the bile ducts can lead to anemia, inflammation, obstruction and cholangitis. The high frequency of non-synonymous polymorphisms found in *Fasciola* spp. genes allows for adaptation and invasion of a broad range of hosts. This is also facilitated by parasite’s excretory-secretory (ES) molecules that mediate physiological changes that allows their establishment within the host. ES contains cathepsin peptidases that aid parasite invasion by degrading collagen and fibronectin. In the bile ducts, cathepsin-L is critical to hemoglobin digestion during feeding activities. Other molecules (peroxiredoxin, cathepsin-L and Kunitz-type inhibitor) stimulate a strong immune response polarized toward a Treg/Th2 phenotype that favors fluke’s survival. Helminth defense molecule, fatty acid binding proteins, *Fasciola*-specific glycans and miRNAs modulate host pro-inflammatory responses, while antioxidant scavenger enzymes work in an orchestrated way to deter host oxidant-mediated damage. Combining these strategies *Fasciola* spp. survive for decades within their mammalian host, where they reproduce and spread to become one of the most widespread zoonotic worm parasites in the world.

## Graphical abstract

**Figure:**
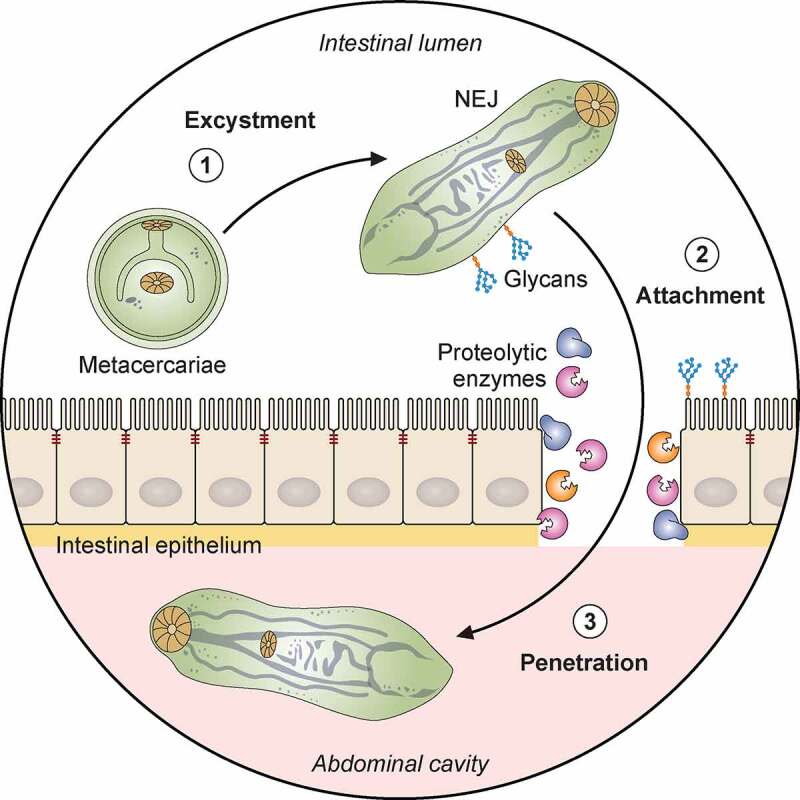

## Introduction

Fasciolosis is a highly pathogenic parasitic disease of humans and their livestock caused by flatworms of the genus *Fasciola*, also known as liver flukes. Infection caused by the liver fluke species, *Fasciola hepatica* and *Fasciola gigantica*, are amongst the most neglected zoonotic diseases, despite their global distribution [1–3]. These parasites are found on all inhabited continents, in more than 70 countries. *F. hepatica* is predominately found in temperate climates, but is also prevalent in the tropical and subtropical countries, including those in the Middle East (Egypt and Iran), South America (Bolivia, Ecuador and Peru) and Asia. *F. gigantica*, the cause of tropical fasciolosis, is primarily found in less-developed regions throughout Asia, Africa and the Middle East [1,4].

Liver flukes are extremely successful parasites and infections have been documented in humans and a range of ruminants, including sheep, cattle, goats, buffalo, camelids and cervids. Less commonly, these parasites infect non-ruminant herbivores (e.g., equids, lagomorphs, macropods, and rodents) [5]. In livestock animals, *Fasciola* spp. infection causes significant morbidity and mortality, and is linked to reduced productivity and fertility and increased susceptibility to co-infections. Together, these contribute to annual economic losses in the order of €2.5 billion worldwide [6–8].

The socio-economic and medical importance of fasciolosis is unquestionable. It is estimated that between 2.6 million and 17 million people are infected with *Fasciola* spp. globally [9,10]. Whilst human cases of *F. gigantica* infection are less common, they have been reported in tropical regions of Asia, Africa, Iran, and Hawaii [5]. However, despite liver flukes being one of the most pathogenic human-infecting trematodes in terms of parasite-associated morbidity, a lack of epidemiological studies and case notification make it difficult to calculate the exact current burden of human fasciolosis. The most recent data, from 2012, estimated the disability-adjusted life-years (DALYs) for this infection at 35,000 per year [10].

Genomic and transcriptomic studies, aligned with functional characterization of somatic and secreted molecules, have allowed a better understanding of *Fasciola* spp. parasite biology, and its relationship with their hosts [11–15]. It is now clear that the different developmental stages of *F. hepatica* express and secrete a set of regulatory proteins, glycans and micro-RNAs (miRNA) that interact with host factors and tissues, mediating physiological changes that favor the establishment of the parasite. The high frequency of non-synonymous polymorphisms found in genes expressed by the flukes has been linked to the ability of *Fasciola* spp. to adapt to such a broad range of definitive hosts [12]. Interestingly, many genes have expanded and diverged to create multi-membered families with very specific, although sometimes overlapping, functions that may also allow the parasite to adapt to different hosts and infection sites during its lifecycle [3,13,14]. Of note, various parasite molecules also display key immunomodulatory properties and often function in conjunction to subvert the host immune responses in favor of the liver fluke parasite [16–22]. Many of these molecules are being exploited to improve diagnostics, and to develop vaccine and drugs to control fasciolosis.

**Figure 1.**
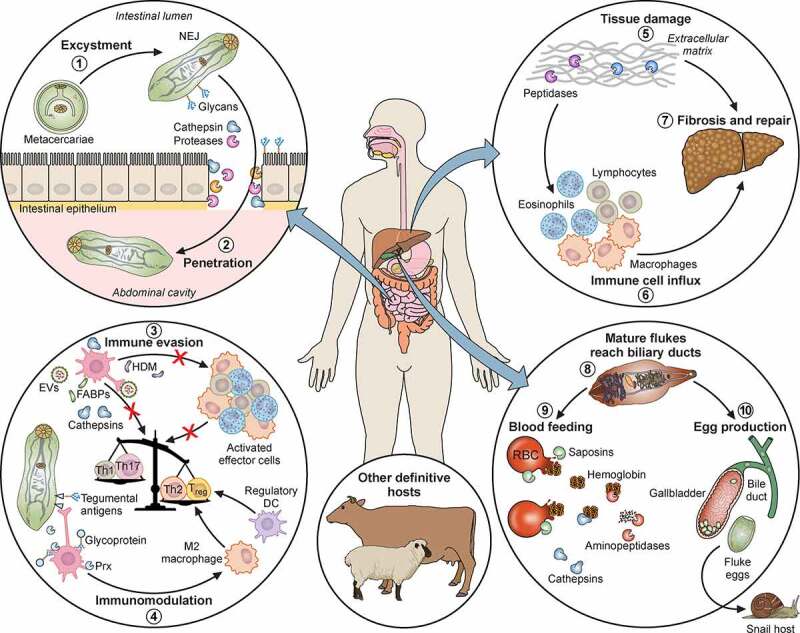
**Pathogenicity of *F. hepatica* within the mammalian host**. 1: The metacercariae of *F. hepatica* become activated by a series of stimuli (CO_2_, temperature, bile salts, reducing conditions, pH) as they pass through the digestive system of a mammalian host. 2: The metacercariae excyst in the small intestine releasing NEJs that attach to the gut wall via surface glycans and penetrate through the intestinal epithelia with the aid of secreted cathepsin peptidases. 3: Once in the abdominal cavity, and throughout its lifecycle, *F. hepatica* expresses a plethora of virulence factors that enable the parasite to evade and modulate the host’s immune response. Many of these factors (Cathepsins; Fatty acid binding proteins, FABP; Helminth defense molecule, FhHDM; Extracellular vesicles, EVs) hamper the activation of host immune cells by limiting their ability to respond to inflammatory stimuli and subsequent capacity to promote antigen specific Th1/Th17- responses that are required to effectively clear infection. 4: In contrast, secreted proteins (Peroxiredoxin, FhPrx; parasite glycoproteins), as well as glycoconjugates on the tegmental surface of the parasite, actively recruit and modulate dendritic cells and M2 macrophages which favor the induction of Th2/regulatory immune responses, creating an immunological environment that benefits the parasites survival. 5: Over a period of days, the NEJs migrate through the abdominal cavity to the liver, where they begin tunneling a path through the connective tissue of the parenchyma, facilitated by parasite secreted cathepsin peptidases (FhCL2, FhCL3) capable of degrading the liver extracellular matrix. 6 and 7: The extensive tissue damage caused by the migration through the liver initiates a wound healing response characterized by the influx of immune cells, and subsequent induction of fibrosis to repair the damage. 8: Flukes reach the biliary ducts of the mammalian host approximately 12 weeks after infection. 9: Blood is a vital nutrient source for the mature parasites in the bile ducts, and they express several proteins related to red blood cell lysis, hemoglobin digestion and metabolism (Saposins, Cathepsins, Aminopeptidases). 10: The acquisition of these extra nutrients from blood allows *Fasciola* to produce thousands of eggs which are shed via the host’s feces that results in the infection of the intermediary snail host, restarting the parasite lifecycle. Adapted from different templates available in BioRender.com (2021). Retrieved from https://app.biorender.com/biorender-templates

Here we review the main mechanisms of virulence and pathogenicity of *Fasciola* spp. parasites. We discuss how surface and excreted-secreted (ES) molecules contribute to infection and establishment within mammalian hosts, in spite of their induction of elaborate immune responses. Specific pathogenesis caused by each lifecycle stage of the liver fluke within the definitive hosts are highlighted (Figure 1), as well as virulence aspects of different isolates and species.

## *Fasciola* spp. lifecycle and biology

As with most trematodes, *F. hepatica* and *F. gigantica* have a complex lifecycle, requiring a vertebrate primary host, in which the liver flukes reproduce sexually, and an intermediate host (aquatic snails in the family Lymnaeidae), in which asexual reproduction occurs [1,23]. Adults of the two *Fasciola* species differ in size and have distinct morphological characteristics. Both are large leaf-shaped worms: *F. hepatica* adults are ~4 cm in length and ~1.5 cm wide, while *F. gigantica* are ~7.5 cm in length and ~1.5 cm wide [24]. These reside in the biliary ducts and gall bladder of the primary host, where they reproduce. *Fasciola* spp. are hermaphroditic, and therefore capable of self-fertilization; however, cross-fertilization between two adult flukes is the most common form of reproduction and contributes to the gene polymorphism observed within these species [12]. The flukes can live for decades within the host and produce up to 25,000 eggs per day per fluke [25]. These eggs are released into the intestine and are passed into the surrounding environment within the feces.

The eggs released into the environment are initially immature but following a period of embryonation develop into a miracidium, a ciliated larva that hatches out of the egg through an opening termed the operculum, and actively seeks and infects a suitable snail intermediate host. The miracidium lifespan (8 to 24 hr) is greatly limited by their glycogen stores, which is their primary energy source. To increase the chances of locating and invading a suitable host snail within this period, the larvae have developed refined chemo-sensorial mechanisms that involve positive phototropism and expression of genes involved in the secretion of pheromones and tissue-degrading metallopeptidases [3,26].

Inside the snail, the parasite undergoes asexual development, going through various stages in sequence, sporocyst, rediae and finally cercariae. Remarkably, during this process, known as clonal expansion, a single miracidium can produce 10 to 700 cercariae [26]. The large number of cercariae emerging from the snail ensures that the lifecycle will progress. The ability of the parasite to survive and reproduce within the snail has been linked to the expression of genes involved in the manipulation of snail innate immune responses. Specifically, transcriptome analysis of the intra-snail stages of *F. gigantica* revealed an up-regulation of genes responsible for innate immune responses, B cell receptor signaling pathways and lymphocyte activation. In addition, increased expression of cathepsin L peptidases has been shown to be associated with their migration and feeding [13,23].

Appropriate temperature and light conditions stimulate the snails to shed cercariae. These large (~250 µm) and motile larvae swim in the water and encyst either on leafy vegetables or at the water surface to form the resistant metacercariae, which is the infective stage for the definitive mammalian host. Encystment occurs in response to changes of environmental conditions (e.g., oxidative stress, UV light, salinity and CO_2_ concentration) that are sensed by the cercariae. Specific proteins expressed on the tegumental surface of cercariae (e.g., aquaporins) have been linked to their ability to detect some of these changes [13].

*Fasciola* spp. infection only occurs when the mammalian host ingest vegetation or water contaminated with metacercariae [1]. In the small intestine, the metacercariae excyst releasing the infectious newly excysted juveniles (NEJs). The NEJs are small (~0.1 mm), active parasites that penetrate the gut wall and can be found in the abdominal cavity 6 to 72 h post infection, depending on the host species [27,28]. This process is fundamental for fasciolosis infection to ensue and marks the beginning of the disease pathology.

Although far smaller and sexually immature, the NEJs already possess most of the structural features of an adult fluke. The outer surface of the juvenile flukes is called the tegument, and its primary function is to protect the fluke from host enzymes and immune attack [29]. It consists of a syncytial layer surrounding the surface of the parasite, and is contained by a plasma membrane covered with a thick carbohydrate coat or glycocalyx. The tegument is a highly functional, metabolically active structure, responsible for absorption of nutrients, synthesis and secretion of substances, osmoregulation, protection, and production of extracellular vesicles (EVs) [19,30]. It has a sensorial role, due to the presence of small spines on the surface, which are also likely to be involved in locomotion [31,32].

The tegument also plays a key role during fluke infection as it actively suppresses the immune response of the mammalian host, allowing the juveniles to develop into adult flukes and continue the lifecycle. Early studies have demonstrated that the tegument is very dynamic, being continuously sloughed off and replaced as the parasite migrates through different host tissues [31,33]. Initially, the NEJ tegumental syncytial layer is dominated by structures referred to as T0 secretory bodies, which change into T1 bodies as the parasite enters the liver parenchyma, and finally to T2 bodies within the fully mature adult parasite [31]. This change of secretory body type is directly associated with the tegument composition and microenvironment the parasite is in, and is considered an immune evasion strategy as the contents of these bodies are released at the apical membrane and added to the glycocalyx [34].

Within one week of ingestion, the parasite crosses the peritoneum and reaches the liver parenchyma by penetrating the Glisson’s capsule. While the juvenile fluke moves through the liver, it grows significantly by feeding on host tissue cells and, eventually, on blood [32,35]. These activities cause most of the clinical symptoms associated with acute fasciolosis. At this stage, the parasite is ~5 mm and mechanical damage arises from abrasion by the parasite tegument, the digestion of the tissue and the action of the suckers as the parasite moves within the parenchyma. These physical actions result in lesions marked with hemorrhagic migratory tracts that are commonly observed in the liver parenchyma. The NEJs quickly manipulate the host’s immune response, preventing the onset of Th1-mediated immune responses by modulating protective innate cells, such as macrophages, and establish a Th2-type of immune response that benefits their survival. After 3 to 4 months, the parasites reach the bile ducts where they develop into sexually mature adults and initiate egg production [36].

**Figure 2.**
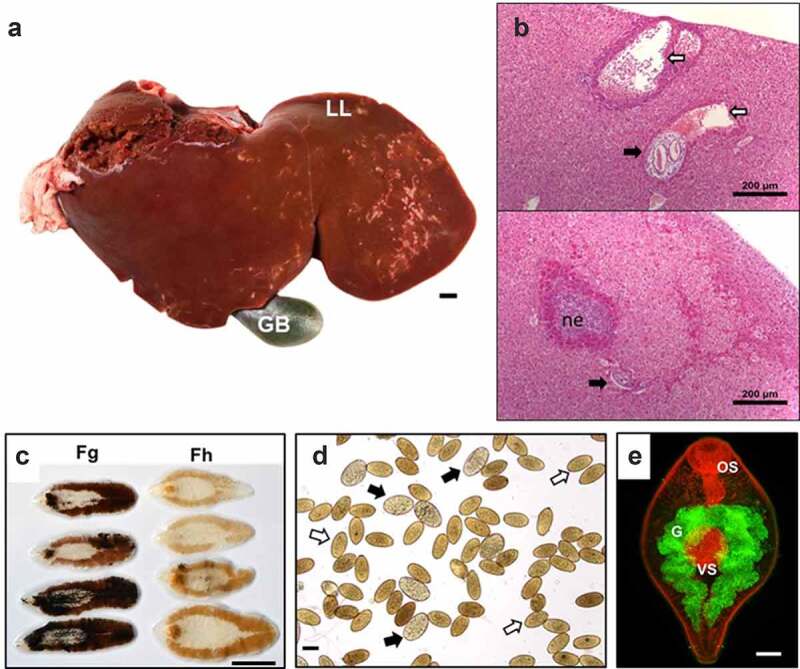
**Life stages of *F. hepatica* and *F. gigantica.*** A: Liver from an infected sheep with acute *F. hepatica* infection (reported by López Corrales et al. [157]) showing gross pathology. The white tracks delineate the tunneling activity of the parasites through the liver tissues, most commonly observed on the left lobe of liver closest to the intestine *in situ* (LL). GB: Gall bladder. Scale bar, 1 cm. B: Microscopical liver pathology shown by hematoxylin and eosin (HE) stained serial liver sections from a mouse infected with *F. hepatica* (Molina-Hernandez and Dalton, unpublished). Top panel: Liver section displaying the migratory tracts (white arrows) formed by the invading *F. hepatica* immature flukes (black arrow). Bottom panel: The damage caused by the migrating parasite (black arrow) is resolved with no visible tracts in the liver and acute necrotic foci (ne) comprised of inflammatory cells (mainly eosinophils and macrophages). Scale bar, 200 µM. C: Adult *F. gigantica* (Fg) and *F. hepatica* (Fh) parasites. Note the typical leaf shape morphology of each species and the size variation. The length-to-width ratio of adult *F. gigantica* is greater than that of *F. hepatica* parasites. *F. hepatica* parasites have a broader anterior end, with defined shoulders, whilst *F. gigantica* is narrower and lacks this definition. Scale bar, 1 cm. D: The differential morphology of eggs from *F. hepatica* (white arrows) and *F. gigantica* (black arrows). The eggs of *F. gigantica* are typically larger, but variation exists in different definitive hosts and thus a considerable overlap is observed. Scale bar, 100 µM. E: The invasive newly excysted juvenile stage of *F. hepatica* has a typical cephalic cone shape. Antibodies to the digestive cathepsin peptidase L3 were used to probe the parasite and highlights their bifurcated gut (g) represented by the green fluorescence. The musculature of the parasite is highlighted by the red fluorescence, that accentuates the oral sucker (OS), ventral sucker (VS) and tegument of the parasite. Scale bar, 20 µM

Adult parasites possess a tough outer tegument surface, with the highly specialized functions discussed above. The spines of the tegument are longer at this stage and help to maintain the position of the fluke within the tissues. The mechanical interactions between hepatic cells and the parasite tegument cause sufficient trauma leading to cell destruction [37]. The spines also facilitate feeding as the parasite uses them for puncturing small blood vessels [27]. *Fasciola* spp. have two suckers, the oral sucker, located at the anterior end surrounding the mouth, and the ventral sucker, both of which cause major tissue damage as the flukes use them to feed, attach to the bile duct walls and to migrate [29,38,39]. Through the oral sucker, food enters the parasite’s bifurcated, blind-ending gut, where it is digested. Nutrients are absorbed through the epithelial layer, or gastrodermis, lining the parasite gut, while the excess undigested material is regurgitated [40] (Figure 2). Therefore, this material, together with a number of excreted-secreted components, is released into the host via the parasite’s mouth [41]. The parasite also secretes many molecules through its tegument, both directly into the microenvironment and packaged into EVs [42,43]. These molecules act at the parasite-host interface, helping the parasite to survive by manipulating the host environment.

A newly laid ovoid egg contains an immature miracidia, and is relatively large (130–150 μm by 63–90 μm), operculated, and yellowish brown in color. The eggs are passed into the bile and might remain in the gall bladder for a certain period. Eventually, they reach the host intestine with the bile during digestion, and from there are excreted with the feces into the environment. In fact, the presence of eggs in feces is one of the most commonly employed tools used for diagnosing patent fasciolosis and the total egg count can give a general indication of the parasite burden [44].

## *F. hepatica* isolates and variation in virulence

Genetic analyses of *F. hepatica* isolates throughout the world have shown that these parasites display high levels of genetic heterogeneity, which has been linked to their wide host range and capacity for rapid adaptation to the host environment and external selection pressures, such as drug interventions [12,45,46]. These genetic differences may also play a role in the parasite virulence. In our laboratory, studies of *F. hepatica* infection have shown that the proportion of parasites that survive to adult stages can vary depending on the isolate. To date, the majority of studies investigating the phenotypic differences between liver fluke isolates are linked to studies of drug resistance, namely triclabendazole (TCBZ) resistance, that is the most commonly used flukicidal treatment of *F. hepatica* in both humans and livestock [47]. The *F. hepatica* isolates most frequently used for these studies are isolates of known TCBZ susceptibility/resistance that have been maintained experimentally for over 20 years [48], including the Sligo (Ireland), Oberon (Australia), Dutch (Netherlands), Cajamarca (Peru) and Rubino isolates (Uruguay) that are considered resistant to TCBZ, while the Cullompton (UK), Fairhurst (UK), Sunny Corner (Australia), and Centro de Diagnóstico e Investigaciones Veterinarias (CEDIVE) isolates (Argentina) are susceptible to TCBZ [49–51].

Analysis of these isolates has shown that they display different phenotypic traits that may influence the virulence and pathogenicity of these parasites. The study by McConville et al. [52] compared the Sligo and Cullompton isolates in sheep, which showed that the resistant isolate from Sligo reached the bile ducts one week earlier compared to the susceptible Cullompton isolate, and also produced eggs two weeks earlier. However, the Sligo isolate flukes were smaller in size, produced fewer eggs and their metacercariae were less infective to sheep when compared to the Cullompton isolate [52]. Similarly, Walker et al. [53] examined differences in cercarial production by the Fairhurst and Oberon isolates and their infectivity in rats. The Oberon isolate demonstrated accelerated egg hatching and production of cercariae, as well as yielding four times the number of cercariae. The resulting metacercariae were also more infectious and displayed an accelerated developmental progression, which was two and half weeks faster when compared to the Fairhurst isolate [49,53]. However, these results must be interpreted with caution as after years of laboratory maintenance these isolates may no longer be representative of field isolates; in fact they display traits such as abnormal spermatogenesis (Sligo) [54] or aspermia, polyploidy (Cullompton) [55] and reduced genetic heterogeneity (Fairhurst) [56] that may affect their virulence and pathogenicity.

Most recently, the study by Hodgkinson et al. [57] highlighted the phenotypic intra- and inter-variation between triclabendazole-susceptible and -resistant clones that were recently propagated from the field. The adult parasites from the TCBZ susceptible isolates (80–280 mg) were larger than their resistant counterparts (20–160 mg). However, despite the parasites being derived from a single miracidium (and therefore considered a clonal line) variation in the size of the parasites obtained from their mammalian host was observed, indicating that the host interactions also play a role in parasite growth and survival. Analysis of the snail-associated stages revealed no demonstrable differences between the isolates in the timing of cercarial shedding or the number of cercariae recovered.

## The snail intermediate hosts of liver fluke parasites

*F. hepatica* utilizes snails of the *Lymnaeidae* family as intermediate hosts, with the favorable species varying between continents and climates. Within Europe, *Galba truncatula* is the predominant intermediate host used by *F. hepatica* [1], likely due to the similar adaptation of both species to require a wet, temperate climate [58]. *G. truncatula* is found throughout Africa, North and South America, and Asia, though other species are also commonly identified as host species in these continents (Table 1) [59–61]. In fact the ability of *F. hepatica* to infect a wide range of lymnaeids that, according the study by Correa et al. [62], are distributed into three main clades (C1 (American species), C2 (Eurasian species) and C3 (Australasian species)) is possibly a determinant for the broader geographic distribution of this species, compared to *F. gigantica*, which have been particularly associated to species in the C3 group.

**Table 1.** Major Species of snails from the Family *Lymnaeidae* infected with *F. hepatica* globally

Continent	Species of *Lymnaeidae*
Europe	*Galba truncatula* *Omphiscola glabra* *Lymnaea palustris*
Africa	*Galba truncatula* *Pseudosuccinea columella* Radix natalensis*
North America	*Fossaria humilis* *Fossaria bulimoides* *Fossaria cubensis*
South America	*Galba truncatula* *Fossaria viatrix* *Fossaria diaphana* *Fossaria cubensis*
Central America	*Fossaria cubensis* *Pseudosuccinea columella*
Australasia	*Lymnaea tomentosa* *Pseudosuccinea columella* *Galba truncatula* *Austropeplea ollula* *Austropeplea viridis*

*Verified by experimental infection [63,68].

A study into the host specificity of *F. hepatica* miracidia identified miracidia-attracting glycoproteins (MAGs) in snail-conditioned water of *G. truncatula*, but not *L. stagnalis*, which play a role in stimulating host-finding responses in the miracidia [64]. Following infection of the snail host, the miracidia undergo the asexual, or clonal expansion, phase of their parasitic lifecycle [65]. When environmental conditions are optimal, the development of *F. hepatica* miracidia to cercariae takes approximately 5 to 7 weeks [66]. The efficiency of the clonal expansion is affected by environmental stresses encountered by the snail; for example, in snails that were exposed to 10 days of desiccation, the number of rediae ranged between 18–25, compared to 43 in the unstressed control [67], suggesting that dry conditions impact the ability of the snails to act as an intermediate host, as well as making conditions challenging for miracidia to survive in the environment. On the other hand, infection with *F. hepatica* has also been shown to have a negative effect on the lifespan and reproductive activity of the snail host, which may play a role in the subsequent prevalence of fasciolosis on pasture [68].

The species of snail host may also impact the number of metacercariae in the environment. An investigation into the suitability of *G. truncatula* and *P. columnella* for metacercarial production suggested that *P. columnella* is the more proficient intermediate host of the two, due to its greater survival rate 30 days post-infection, and ability to produce two-fold higher numbers of cercariae than *G. truncatula* [69]. While multiple species of snails are able to act as the *F. hepatica* intermediate host, the host species may impact the lifecycle of *F. hepatica*, as the composition of ES products from NEJs can change depending on the intermediate snail host. A study comparing the secretome of NEJs from *L. viatrix* and *P. columnella* identified five proteins that were unique to NEJs that had used *L. viatrix* as the intermediate host, and thirty nine that were unique to *P. columnella*-derived NEJs [65]. It is important to note that this study used miracidia from two different *F. hepatica* isolates, and therefore the differences in the NEJs secretome may not necessarily be solely attributed to the snail host species.

Unlike *F. hepatica*, the lifecycle of *F. gigantica* tends to occur in regions with defined wet and dry seasons. In areas where *F. gigantica* is most common, various *Radix, Lymnaea* and *Galba* snail species act as intermediate hosts, all of which rely on the presence of well-oxygenated, slow-moving water for survival [62,70,71]. Even so, in regions of varying altitudes, where the environment supports the survival of several snail species permissive to infection with either species, such as in Pakistan and Tanzania, overlaps in the distribution of *F. hepatica* and *F. gigantica* occurs [72,73]. The snail species susceptible to infection with *F. gigantica* have varying capacities to adapt to dry seasons and may undergo aestivation in mud until suitable conditions return the following season [70]. The susceptibility to drying affects the survival of parasite stages within the infected snails and, consequently, the availability of infective metacercariae in the environment. The development of *F. gigantica* cercariae in infected snails is impeded at temperatures below 16°C, which is higher than the 10°C limit typically observed with *F. hepatica*, and demonstrates an adaptation to warmer climatic conditions [70,74].

Snails infected with *F. gigantica* produce a higher proportion of floating metacercariae than those infected with *F. hepatica* (17–35% vs 4–7%, respectively) [75,76]. This finding has important implications for routes of transmission in both livestock and human populations, and suggests that contaminated water sources may play a more significant role in the transmission of *F. gigantica* than *F. hepatica*. Once in the environment, the survival of *F. gigantica* metacercariae is a function of humidity, temperature, pH, and exposure to direct sunlight. The metacercariae of *F. gigantica* are more tolerant to higher temperatures than *F. hepatica* and will remain viable between 2–35°C when adequate humidity is maintained [77–79]. Exposure to direct sunlight results in 100% mortality of *F. gigantica* metacercariae within eight hours, compared to only two hours for *F. hepatica* [80,81]. Transcriptome analysis of *F. gigantica* cercariae have revealed a shift in gene expression toward decreased metabolic processes and nucleotide synthesis as they mature into recently encysted metacercariae, and suggests that their low metabolic rate is maintained by pH regulation and an avoidance of autolysis via an associated reduction in endopeptidase activity [3]. Each of these factors play an important role in the maintenance of *F. gigantica* in the environment and influence the various routes of transmission to mammalian hosts.

## Pathogenicity of *Fasciola* spp. in their definitive hosts

### Livestock

*Fasciola* spp. are zoonotic parasites that infect primarily domestic livestock animals, most commonly sheep, goats, cattle and buffaloes. Infection manifests in three phases: acute, sub-clinical and chronic. In sheep, acute disease is often first detected by sudden death of up to 10% of the flock, usually due to high levels of blood loss from physical damage to the liver [82]. Symptoms of acute infection in sheep can also include reluctance to run due to abdominal pain, lethargy and reduced appetite for grazing. Acute fasciolosis in sheep can be complicated by secondary infection of the liver by *Clostridium noyvi*, resulting in clostridial necrotic hepatitis [83]. Sub-clinical disease is slightly more delayed than acute disease and presents as hemorrhagic anemia. Chronic fasciolosis in sheep presents as failure to thrive due to low body weight and poor quality fleece, and also severe swelling under the jaw known as bottle-jaw [82]. While *Bos taurus* cattle usually build a degree of immunity to infection with *F. hepatica* infection, this is not observed in sheep [83].

Studies in sheep have revealed the impact of *F. hepatica* on the serology profiles of infected animals. Significantly lower levels of total protein, albumin, glucose, triglyceride, cholesterol and high-, low- (LDL) and very low-density (VLDL) lipoproteins were detected in infected sheep compared to uninfected controls, while the activity of aspartate aminotransferase (AST), alanine aminotransferase (ALT), γ-glutamyl transferase (GGT) and lactate dehydrogenase (LDH) were significantly higher in infected animals [84]. These differences were still detectable 28 days after drug treatment, but no significant differences between the control and treated groups were observed 56 days post-treatment [84]. In another study, infected sheep had significantly higher levels of GGT, and total and direct bilirubin compared to uninfected controls [85]. These changes in the serology profiles of infected animals are associated with liver damage that causes liver enzymes to leach into the blood, and with the establishment of adult flukes in the biliary ducts. While the cellular response of sheep is consistent regardless of the level of infection, the degree of eosinophilia increases in line with the level of infection [86].

Acute disease is rarely seen in cattle, instead, the animals tend to develop chronic disease if they acquire a particularly heavy infection [87]. However, cattle and buffaloes calves exposed to heavy infections may suffer from acute fasciolosis and death [88]. In the event of extremely high fluke burdens, clinical disease may occur as a result of the extensive damage to the liver caused by migrating juvenile flukes [66]. Liver fibrosis in infected cattle is far more severe than that in infected sheep [89] and there is a positive correlation between the extent of liver fibrosis and the number of adult fluke recovered at necropsy [90]. An assessment of cattle carcasses in Uruguay identified a positive correlation between *F. hepatica* infection and a reduction in carcass weight, with the degree of weight loss being more significant in younger animals aged less than 30 months [91]. A similar study conducted in Brazil comparing the weight of cattle following *F. hepatica* infection showed that weight loss of up to 11% can occur in infected animals compared to uninfected animals [92]. In agreement with these studies, infection with *F. hepatica* was shown to reduce weight gain by up to 10 g per day, and delay slaughter by up to 2 weeks, compared to uninfected animals [93].

The clinical symptoms of fasciolosis in cattle generally include weight loss and diarrhea, while dairy cows may also present with reduced milk production and fertility [89]. In high yielding dairy herds, milk production may be reduced by up to 15% [94], representing a financial loss of approximately £300 per cow per annum [95]. As well as a reduction in milk production, infected dairy herds show a significant average reduction in milk protein and fat content of 0.06 kg compared to uninfected herds [96]. *F. hepatica* infection in dairy cattle is also associated with an increase of 4.69 days in the time between calving and conception [97]. Calves born to infected cows may be weak and sickly due to receiving inadequate nutrition from their mothers [89].

Similar to sheep, studies in cattle have identified the effects of *F. hepatica* on the serological profiles of infected cattle. Infected animals show significant increases in the liver enzymes AST, GGT and alkaline phosphatase (AP) compared to uninfected animals [98], reflecting the damage to the liver caused by the flukes. A study in Argentina found that cattle infected with *F. hepatica* showed significant increases in leukocytes, eosinophils, GGT, gamma globulin and total protein in blood and serum samples compared to uninfected controls, which is indicative of cholestasis and liver inflammation, dysfunction and necrosis [99].

Postmortem examinations of sheep and cattle carcasses can confirm liver fluke infection by the presence of lesions and tracks in the liver and eggs in the gall bladder. However, the pathology of fasciolosis could be greatly prevented by early diagnosis, which allows for appropriate anthelmintic treatment before the parasite reaches the liver and bile duct of the host. Unfortunately, most diagnostic methods available have drawbacks and are not ideal for detection of the immature stages. Fecal eggs counts (FEC) are only useful to detect patent infections and have poor sensitivity with low burden infections [100]. Enzyme-linked immunosorbent assays (ELISAs) such as the coproantigen-ELISAs offer an alternative method of diagnosis from FEC, but they are limited in detecting infection inside the pre-patent period [101,102]. Of the methods mentioned in Table 2, only serological ELISAs were proven to detect specific anti-*F. hepatica* antibodies as early as ~3 weeks post-infection [103,104]; however, this method cannot distinguish between new and historic infections. Diagnosis can be complemented by considering nonspecific symptoms, namely increased liver enzymes in serum (i.e., ALT, AST, GGT, LDH and AP), anemia and decreased serum albumin levels. Elevation of specific hepatic enzymes in host circulation has been demonstrated to be synchronous with the pre-patent (AST and ALT) and patent (AP) phase of infection [105]. Moreover, currently only DNA-based diagnostic methods can reliably differentiate between infections with *F. hepatica* and *F. gigantica* [106].

**Table:** 

**Table 2**. Main diagnostic methods for fasciolosis.			
**Method**		**Target**	**Applicability**
**Coprological**	FEC (Microscopy) [107]	Eggs in feces	Patent infection
	Coproantigen ELISA [108]	FhCL1 antigen in feces	Late pre-patent – patent infection
**Serological**	ELISA [104,109–111]	FhCL1 antibodies in serum or milk	Early pre-patent – patent infection
	Lateral flow [112]	FhCL1 antibodies in serum	Early pre-patent – patent infection
**Molecular**	PCR [106,113]	Internal transcribed spacer 1 (ITS1), internal transcribed spacer 2 (ITS2) and large subunit (LSU) rDNA in feces	Late pre-patent – patent infection Differentiation of *F. hepatica* and *F. gigantica*
	LAMP [114]	ITS2 rDNA in feces	Late pre-patent – patent infection
Fecal egg count (FEC); Enzyme Linked Immunosorbent Assay (ELISA); Polymerase chain reaction (PCR); Loop Mediated Isothermal Amplification (LAMP)			

### Humans

Liver flukes have been infecting humans for over 5000 years [115,116], yet it was not until 1760 that the first case was described during the autopsy of a female in Germany [117]. But even up to the beginning of the 1990s, fasciolosis was still not considered an important disease of humans. That changed in the early 1990s when Hillyer [118], Bjorland and colleagues [119] described a very high prevalence of fasciolosis amongst the native Aymaran population of the Bolivian Altiplano. The Altiplano corridor stretching from Bolivia, through Peru to Ecuador still represents the region of highest endemic fasciolosis in the world. Remarkably, since the introduction of *F. hepatica* from Europe, sometime during the last 450 years, the parasite has adapted well to the high altitudes of >13,000 ft and to the local intermediate hosts. Varying levels of prevalence, from 5.9% to 70%, are found sporadically throughout the region depending on the hydrology, geography, snail host distribution and levels of animal infection [120,121]. A greater focus on the emergence of human fasciolosis over the last 30 years has discovered major endemic regions in China, South-East Asia (such as Vietnam), Egypt, Turkey and Northern Iran [10,122–125], and that outbreaks or cases occur across 80 countries where animal fasciolosis is also present [10,126]. Consequently, fasciolosis has been recently recognized as an important neglected zoonotic disease of humans by the World Health Organization [10]. The emerging importance of human fasciolosis has spurred the publication of several excellent detailed reviews over the last five years [9,46,126].

Infection in humans is mainly acquired following ingestion of edible aquatic vegetables and plants, which vary depending on the region [46,121,127,128]. Contaminated vegetables may be sold in local markets that are distant from the source of parasites and snails such as that found in outbreaks in some towns in Northern Iran [129,130]. As many metacercariae float rather than adhere to vegetation the drinking of water carrying parasites or the consumption of vegetables washed in contaminated water can be another means of infection [46,131]. Sporadic cases in Europe and elsewhere are often associated with the eating of wild watercress foraged from the side of rivers [132,133]. Not surprisingly therefore, outbreaks tend to be local and familial [134]. In highly endemic regions, such as Bolivia, Egypt and Vietnam, children are more in danger of the consequences of disease (anemia, liver damage, impaired cognitive development) and appear to be more susceptible to infection. While Parkinson et al. [135] found no significant association between infection levels and sex in their studies in Bolivia, a survey of >21,000 children in Egypt found a higher prevalence in females as well as a greater number of eggs in their stool samples [136].

The pathology of fasciolosis in humans depend on several variables, including fluke species and isolates, parasite burden and host biology (e.g., immune status, age, nutrition). The clinical manifestations of fasciolosis caused by *F. hepatica* and *F. gigantica* are considered the same, although the larger size of the latter may result in a greater chance of biliary obstruction [137]. Disease pathology is mostly associated with the trauma caused by the immature flukes burrowing through the intestines and liver parenchyma and this correlates with the level of infection [88]. Low level infections may be asymptomatic or include mild symptoms at the acute stages but can progress to a serious chronic inflammatory situation at a later stage [36]. However, in general, acute infection is characterized by vigorous host immune responses directed to the invasive parasites and their antigens, and may result in fever, nausea, abdominal pain, hepatomegaly, weight loss, anemia, transitional eosinophilia and elevation of liver enzymes [138,139]. The migrating parasites damage tissues and blood vessels causing large subcapsular liver hematomas that can be life-threatening [36,140,141]. These symptoms can last for 2 to 4 months but in endemic regions repeated infections result in overlapping of acute and chronic symptoms [120,142].

Although chronic infections are often asymptomatic, they may be associated with signs of biliary obstruction, abdominal pain and fatty food intolerance [88]. These can take months or even years to manifest themselves. Over time adult parasites cause damage to the bile duct with their spines when they move along the biliary tree. They also secrete many antigens and puncture the bile duct walls to gain access to blood which ultimately leads to hyperplasia of the bile duct epithelium and chronic inflammation, and eventually cholangitis and cholecystitis [121,143]. The pathological signs of human fasciolosis are varied and are dependent on overall parasite dose [1], but include fibrotic lesions and micro-abscesses, and necrotic tracts within the liver parenchyma surrounded by immune cells including eosinophils, consistent with that observed in sheep and goats [144] (Figure 2). Ectopic fasciolosis, whereby parasite migrate to tissues other than the liver (e.g., lungs, intestines, brain), has been described in humans but is not the norm [145].

While diagnosis of human fasciolosis in endemic areas is relatively straight-forward because it is suspected and recurrent, in areas where it is not common difficulties arise in diagnosis because the complex development and migration of the parasites ensure a changing face of symptoms. Moreover, symptoms are generally nonspecific in nature and can be easily mistaken for other diseases, especially those of the liver, and range from mild to severe [146,147]. Patients usually present with a variety of indicators including fever, headache, fatigue, chills, sweats, abdominal pain, epigastric discomfort, rashes and may also suffer from anemia and weight loss [142]. Clinical hallmarks includes elevated levels of liver enzymes (e.g. AST, AP in acute stages and GGT in chronic stages) and high peripheral eosinophilia [9,105,148]. In the clinic, computerized topography (CT) imaging can identify hypodense liver nodules and lesions, branching tracks associated with parasite migration and bile duct enlargement in the chronic infection [148–150]. Liver biopsy may show hepatitis, inflammation, acute necrosis and eosinophilia in portal and sinusoidal spaces, but is unlikely to detect parasites in tissue [148]. Ultrasonography of the abdomen could detect intra-hepatic bile duct enlargement. In a recent study, live worms were visualized in the major duodenal papilla by endoscopic retrograde cholangiopancreatography and then extracted for identification [151].

Serological examination, especially enzyme-linked immunosorbent assay ELISA, has proven an important adjunct for diagnosis, but these tests are not routinely available in the clinic. In the USA, the Center for Disease Control (CDC) recommends an immunoblot assay with *Fasciola* saposin antigens (FhSAP2) [152], whereas in Europe ELISAs that exploit parasite-secretory antigens or cathepsin L peptidases (FhCLs) are used [132,153]. Following treatment with triclabendazole (Novartis, 10 mg/kg on day 1 and 2), which is effective against both migratory and bile duct parasites, symptoms and clinical signs generally resolve within 1 to 2 months, although a follow-up treatment may be required [121,154,155]. The emergence of triclabendazole-resistant parasites in livestock globally is of concern for the treatment of human infection with at least one study indicating reduced treatment efficacy in an endemic area of Peru [156].

## Mechanisms of pathogenicity of *Fasciola* spp. parasites

### Means of infection and excystment

The ability of *Fasciola* spp. cercariae to encyst on vegetation and in water allows for the contamination of both food and drink, and increases the chances of infection of both animals and people. Moreover, encysted metacercariae are hardy and can survive for long periods in the environment, which contributes to the parasite survival and virulence [3,158]. Indeed, cysts were observed to remain infective after being dried or exposed to 1% corrosive sublimate solution for 24 hr or to 50% alcohol for 2 hr [159], which is mainly due to the resistant walls that enclose the metacercariae. The four layers that form the cyst are composed of tanned protein, mucoprotein, acid mucopolysaccharide, and keratinized protein embedded in a matrix consisting of protein and lipid, which together provide structural rigidity and protection against desiccation, toxic substances and attack by bacteria and fungi [159]. Furthermore, contrary to earlier assumptions that the metacercariae are dormant, transcriptional analysis revealed that these stages are metabolically active, transcribing genes involved in the regulation of redox metabolism (FhPrx; superoxide dismutase, FhSOD; and, FABP), pH and endopeptidase activity (cathepsin L and B peptidases, and legumain) [3]. This significant metabolic activity of metacercariae, however, is associated with their reduced infectivity of older cysts and limited longevity [158,160,161].

Infectivity of the metacercariae is influenced by a number of factors including the definitive host, parasite isolate, climatic conditions, seasonality, snail host species and larval stage of development in the snail [49,69,158,161]. As the metacercariae are the infective stage of *Fasciola* spp., the progression of the disease is associated with the dose of metacercariae ingested, the isolate and the host species. Nonetheless, in general, after ingestion of the metacercariae by the mammalian host, excystment occurs within a few hours and the NEJs immediately begin boring through the wall of the host intestine.

The excystation process is complex. In the stomach, host acid peptidases remove the outer cyst layer, initiating the active emergence phase. Activation of the larvae within the inner cyst occurs in the stomach, and is stimulated by high CO_2_ conditions and a temperature of approximately 39°C. Within the duodenum, escape of the NEJs from the metacercarial cyst is prompted by bile salts and reducing conditions (Figure 1) [3,158]. Genes involved in the expression of cell adhesion molecules such as intergrins and cadherins, as well as cytoskeletal proteins such as talins, are up-regulated in the metacercariae relative to the other lifecycle stages. Although the role of these molecules during this stage of infection is yet to be characterized, it is possible that they enable the metacercariae to sense the environmental changes necessary to start the excystment process [12].

Recently, Cwiklinski et al. [13] reported the up-regulation of two genes associated with the response to lipopolysaccharide (LPS) in the metacercariae stage. Considering the environments the metacercariae must endure (pasture and then the host gut), these proteins could have important roles in protecting the cyst. Moreover, although not yet characterized, it is possible that these proteins function to stop bacteria entering the host blood stream as the NEJs burrow through the host intestinal wall, hence preventing local pro-inflammatory responses that could damage the larvae and block infection [13].

### Invasion and migration in the mammalian host

**Figure 3.**
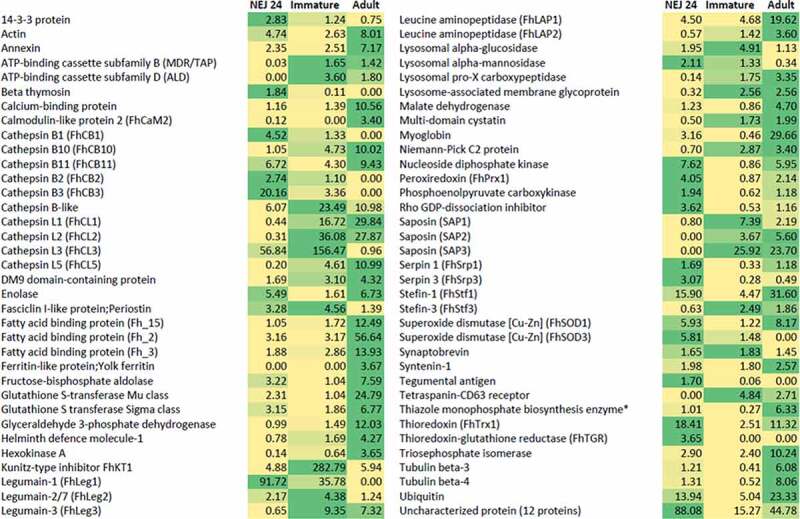
**The profile of proteins secreted by the three main life cycle stages migrating through the host**. The proteomic data for the newly excysted juveniles 24 h post-excystment (NEJ 24), the immature fluke 21 days post-infection (Immature) and the adult parasites (Adult) is extrapolated from Cwiklinski et al. [13], Cwiklinski et al. [197] and Murphy et al. [183], respectively. The protein abundance across the three stages, represented by the emPAI values detailed, is highlighted from low to high abundance by the yellow to green color scale, respectively. Proteins that are secreted in multiple isoforms, such as the cathepsin L peptidases, have been grouped together. The 12 uncharacterized proteins are shown as one group. *Abbreviated name for 4-methyl-5(B-hydroxyethyl)-thiazole monophosphate biosynthesis enzyme

Post-excystment, the parasites enter a new phase of their infective cycle, migrating through the tissues of a mammalian host. The NEJs must transverse the wall of the small intestine rapidly, as their viability decreases significantly while they remain in the gut. They rapidly burrow through the gut wall and have been observed in the abdominal cavity of multiple experimentally infected hosts’ hours after ingestion [162,163]. There is a marked change in the parasite’s metabolism as it migrates through the host, from aerobic energy metabolism to anaerobic metabolism, highlighted by changes in the expression of enzymes related to these pathways at different stages of infection Figure 3[13,164]. Concomitantly, to penetrate through the intestinal tissues, NEJs secrete a range of stage specific peptidases and proteolytic-related proteins, key virulence-associated factors required to breakdown components of the extracellular matrixes (ECM) that hold tissues together (Figure 3).

The high expression and subsequent excretion-secretion of five cathepsin cysteine peptidases, namely cathepsin L3 (FhCL3) and cathepsin B peptidases (FhCB1, FhCB2, FhCB3, and FhCB9), contribute to the rapid excystment of the metacercariae and subsequent invasion of the host by the NEJs [13,165–168]. In addition, three legumains are present within the cysts at twice the abundance of the cathepsin L and B peptidases, and are likely essential to speed up the *trans-*processing of the zymogen forms of the cathepsins to active peptidases [41,167,169]. Unlike traditional cathepsins, *F. hepatica* cathepsin peptidases have acquired substitutions in their active site that enable these enzymes to degrade a diverse range of host ECM macromolecules including collagen, fibronectin and lamins [170,171], which is a common evolutionary adaptation shared by other invasive parasitic helminths [172]. These peptidases were shown to play a pivotal role in the virulence of the parasite as RNA interference experiments targeting the cathepsin L and B genes significantly impaired the ability of the NEJs to penetrate through the intestinal wall [173,174].

More recently, the interaction of NEJs with the intestinal epithelia has also been shown to be implicated in initiating migration. Oligomannose-type N-glycans identified on the surface of NEJs play an integral role in this interaction, mediating contact between the parasite and the intestinal epithelia that signals NEJs to up-regulate essential factors required to penetrate through the intestinal wall [168,175]. Furthermore, blocking of these surface glycans significantly impaired the capacity of NEJs to attach and migrate through the intestine, highlighting the importance of tegumental carbohydrates in the pathogenesis of the parasite during the establishment of infection [176].

Once in the abdominal cavity, the NEJs migrate toward the liver. However, in naïve animals, there is generally very little evidence of pathology associated with the migration of the NEJs during this early invasive stage [177–179]. Several studies attribute the lack of host responses to the broad repertoire of virulence-associated factors expressed by NEJs, which often are involved in subverting the hosts’ capacity to elicit an immune response to stop invasion [180]. It was suggested that this host immunomodulation occurs soon after excystment as NEJs begin interacting with the intestinal epithelia and down-regulating proteins related to ubiquitination [168], a critical process required for intracellular signaling and subsequent triggering of the immune responses [181]. Whilst the mechanism by which NEJs suppress ubiquitination remains unclear; the juvenile parasites express a plethora of molecules that are known to antagonize immune cell-signaling cascades.

As mentioned above, NEJs slough off and rapidly replace their outer tegmental surface as a means of evading surveillance and damage by the host immune response [34,182]. However, ES products and EVs released from the tegument also contribute to such escape. Several of the proteins identified in the secretome of NEJs lack a signal peptide and, thus, it has been suggested that the parasite uses EVs as an alternative route to deliver them into the host tissues where they have been observed to interact and modulate host cells [183,184]. Recent studies demonstrated that the EVs’ cargo contains not only proteins and glycoconjugates, but also specific miRNAs that inhibit mitogen-activated protein kinase (MAPK) signaling in macrophages and their subsequent capacity to respond to inflammatory stimuli [185]. Fromm et al. [43] also showed that these parasite EVs modulate the innate immune system of the host via specific miRNAs, which are enriched in EVs and taken up by host cells. In fact, the distribution of specific miRNAs was shown to vary greatly between EVs released by juveniles and adults stages, which might reflect the levels of interaction each lifecycle stage has with the host.

The peritoneal cavity is a critical location in the development of *F. hepatica* infection. It is not only the route of migration, but also the site where the parasite begins actively inducing an immunological environment that benefits their survival and likely plays a critical role in determining the ultimate outcome of the infection [186]. In this environment, the NEJs have to deal with the rapid cellular and innate immune responses that are alerted by their presence. The parasites are thought to respond to this pressure and avoid, amongst other host defenses, the oxidant-mediated damage by increasing the expression of an array of antioxidant scavenger enzymes [187]. However, Ruiz-Campillo et al. [188] noted that NEJs in the peritoneum do not increase the expression of inducible nitric oxide synthases (iNOS), responsible for producing the toxic nitric oxide, indicating that the parasite anti-oxidant proteins are not involved in antioxidant defense at this stage, but in modulation of the immune response.

Several animal studies have demonstrated that experimentally-acquired or naturally-occurring resistance to *F. hepatica* infection requires a Th1-type immune response. In contrast, in susceptible hosts the dominant immune response elicited by the liver flukes is strongly polarized toward a T regulatory/Th2 phenotype [189,190], which is thought to help repair the damage caused as the parasites migrates through tissues. Several molecules expressed and excreted/secreted by NEJs have been shown to actively contribute toward establishing this regulatory/Th2 immunological environment, including the antioxidant enzyme FhPrx1, which induces the polarization of peritoneal macrophages toward an M2-like phenotype (Figure 1) [189,191]. Similarly, secreted glycoproteins and glycoconjugates on the tegumental surface of NEJs are also heavily implicated in the recruitment and modulation of dendritic cells (DCs) and M2-macrophages in the peritoneal cavity. These cells secrete cytokines that favor the induction of Th2/regulatory immune responses [19,20,175].

The migration of NEJs in the abdominal cavity can last for up to a week, but some immature flukes have been observed in the liver parenchyma as early as 3 days post infection [162]. However, not all NEJs reach the liver. Many experimental infection studies have demonstrated that infectivity is always below 100%, whereby the number of mature flukes recovered in the hepatic canals is never equal to the number of infective forms administered [160,192]. In part, this is due to a portion of NEJs failing to excyst or penetrate the gut wall. Moreover, a thickening of the external fibrous layer of the liver occurs in response to damage induced by NEJs that have already penetrated the liver parenchyma, rendering it less amenable to late-arriving NEJs or subsequent infections. Therefore, either because of their inability to penetrate this hardened tissue or due to the unfavorable environment and less readily available nutrients as a result of liver damage, some NEJs fail to develop into mature adult flukes. These unsuccessful NEJs may migrate through the diaphragm instead, causing occasional hemorrhagic tracts and necrotic lesions in the thoracic cavity [25,193]. This is more evident as the infective dose is increased, resulting in a crowding effect that lowers overall percent take of infective doses [192,194].

### Mechanism of migration through the liver parenchyma

Liver pathogenesis associated with fasciolosis arises from a complex interplay between host and parasite. It is instigated by a combination of mechanical and enzymatic damage caused by the parasite’s migratory and feeding activities in the parenchyma, in addition to the host’s inflammatory immune responses aimed at repairing the ensuing tissue damage, and eliminating the parasite [195].

#### Liver fluke-induced liver damage

Damage to the liver parenchyma ensues following penetration of the Glisson’s capsule, whereby the NEJs migrate into the liver by tunneling a path through the connective tissue between the fibrillary collagen bundles [196]. In the case of infection with *F. hepatica*, this damage continues over the course of approximately eight to 10 weeks, as the parasite continues to burrow through the liver tissue. It occurs by mechanical means aided by the oral and ventral suckers, as illustrated by the presence of hepatic cells inside the suckers [38,138], and by the proteolytic actions of the parasite secreted enzymes such as cathepsin L peptidases (FhCL2 and FhCL3) [197–199]. These cathepsin L peptidases are abundantly secreted by the liver migratory stages (Figure 3) [197] and display potent collagenolytic activity capable of degrading insoluble collagen, which leads to the destruction of the liver ECM and facilitates passage of the parasite [200].

During the liver migratory phase, the liver fluke’s digestive system undergoes rapid development [201]. This allows the parasite to actively feed on host tissue and blood rather than relying on endogenous glycogen stores, expediting growth and development. In addition to FhCL2 and FhCL3, at 21 days post-infection the parasites begin to secrete an array of peptidases involved in the digestion of blood, reflecting the transition to obligate blood feeding (Figure 3) [197].

#### Liver repair mechanisms

The liver is a unique organ with the capacity to not only repair but also regenerate following injury [202,203]. Penetration of the liver by parasites initiates a wound healing response, which in sheep is characterized by an influx of lymphocytes, macrophages and eosinophils, and the induction of fibrosis to repair the damage [186]. This leads to the subsequent formation of visible fibrotic hepatic tracts and granulomas, which are correlated with increased levels of Foxp3 + T regulatory cells that may play a role in reducing tissue pathology [204], overexpression of regulatory cytokines (IL-10 and TGF-β), and pro-inflammatory cytokines (TNF-α and IL-1β) [205]. However, in the case of liver fluke infections in the field, animals can be continually re-infected, perpetuating the damage and wound-healing repair and resolution mechanisms and eventually compromising overall liver function (Figure 1) [186,206].

The delicate interplay between mammalian host and parasite results in different severities of clinical signs and liver pathology depending on the host species. In sheep and goats that are highly susceptible to reinfection, the migratory tracts are surrounded by an infiltration of immune cells as described above, resulting in inflammation and the formation of granulomas [186]. In contrast, infection in cattle causes more extensive fibrosis and less visible tracts, which is thought to play a role in the partial resistance to reinfection, but can progress to cirrhosis of the liver in severe cases [90].

#### Evasion and modulation of host immune responses

During the migratory phase, the liver fluke parasites employ several methods to escape the host immune response to ensure their survival. In addition to evading the influx of immune cells directed to their migratory path by rapidly migrating through the liver parenchyma [37,207], the parasites stimulate polarization of the host immune responses toward a Th2/T regulatory phenotype.

The secretome of the immature 21-day parasites found in the liver is dominated by cathepsin peptidases and their inhibitors [197]. In addition to their role in tissue degradation and feeding, the cathepsin L peptidases have also been shown to cleave the Fc domains of immunoglobulins, preventing antibody-mediated attachment of host immune effector cells and complement activation [208,209], both of which are protective mechanisms required to clear the parasite in some resistant animal species [210]. Moreover, cathepsin-L peptidases are internalized by host immune cells and degrade the pathogen recognition receptor Toll-like Receptor 3 (TLR-3), preventing TRIF-dependent signaling that is crucial for the development of Th1 inflammatory responses that are harmful to the parasite’s survival [211]. This is complemented by the cathepsin peptidase inhibitor, Kunitz type inhibitor, FhKT1, which has also been shown to prevent the development of Th1 and Th17 responses by regulating LPS-stimulated dendritic cells in an IL-27 dependent manner [212]. The liver migrating parasites also secrete several proteins that play a role in the reduction of pro-inflammatory responses, including the FhHDM [213,214] and several FABP; Fh2, Fh3, Fh15 [215–218]. In addition, interaction with the glycans associated with the parasite tegument and ES proteins has been shown to regulate the maturation and function of CD11c+ dendritic cells that are recruited to the liver, where they drive Th2/T regulatory polarization of the host immune response [219].

The ES products also play a role in moderating the eosinophils recruited to repair and regulate liver damage during fasciolosis [220], by inducing apoptosis in the liver-associated eosinophils [221]. This is comparable to the *in vitro* apoptotic effects of the ES proteins on peritoneal eosinophils and macrophages [222–224]. Furthermore, transcriptional analysis revealed that pro-apoptotic signals are increased in peripheral blood mononuclear cells recovered from *F. hepatica* infected sheep and cattle [225,226]. The specific proteins that bring about this cellular apoptosis have yet to be characterized, and further studies are required to determine whether similar parasite proteins are involved within the different immune compartments of the infected animal.

Recently, *in silico* analysis has revealed that *Fasciola*-specific miRNAs may also play a role in regulating the recruitment and functionality of key innate immune cells, targeting several host genes related to dendritic cells, neutrophils and eosinophils [227]. Consistent with their effect on the host immune cells, *in vitro* analyses have shown that the *F. hepatica* ES proteins can also have a direct effect on the liver hepatocytes, reducing their metabolism and overall survival [228–230].

Fasciola *spp. associated oxidative stress in the liver*

A characteristic feature of fasciolosis are the high levels of oxidative stress associated with the pathology caused by the migrating parasites and the resulting host damage repair mechanisms, which both host and parasite must contend with [231–233]. The host’s first line defense responses quickly become overrun, and down-regulation of proteins such as SOD and catalase occurs concomitantly to increasing levels of oxidative stress associated with the parasite-induced liver fibrosis [234–237]. This results in a switch to glutathione thiol-dependent based antioxidants, with increased transcription of glutathione peroxidase (GPx) and glutathione S transferases (GSTs) by the host [197]. In response, *F. hepatica* expresses and secretes an abundance of FhGSTs, FhTrx and FhSOD during the liver stage, indicating that the parasite utilizes a combination of thioredoxin and glutathione thiol-dependent antioxidant systems to counter-attack the levels of damaging reactive oxygen species (ROS) in their environment [197]. Similarly, survival of the parasite within the bile ducts also depends on its ability to eliminate ROS (i.e., hydrogen peroxide and superoxide) generated by host immune effector cells such as macrophages and eosinophils. Consequently, an up-regulation of FhTGR, FhTrx, and FhPrx is also observed within the adult parasites [13,30,183,210].

Consistent with many genes within the *F. hepatica* genome, the repertoire of genes involved in anti-oxidant defenses have undergone gene duplication and expanded into larger gene families [12]. This has resulted in FhTrx and FhPrx members with extended functions that play a role in the parasite-host interplay. Although these molecules generally function in an interdependent manner, by FhTrx reducing and activating FhPrx, recently it was proposed that *F. hepatica* Prx1 and Trx1 could also work autonomously [187]. Moreover, both FhPrx1 and FhTrx1 may contribute to host immune evasion by inducing Th2 immune responses [187,189,191].

The bile-associated parasite stages also produce and secrete high levels of proline, which has been shown to be involved in bile duct hyperplasia during the chronic phases of infection [238–241]. Proline may also play an important role in stabilizing the antioxidant enzymes, in addition to its role in direct scavenging of ROS [197,242,243].

### Adult flukes in the bile ducts

*Fasciola* flukes reach the biliary ducts of the mammalian host approximately 10 to 12 weeks post infection, marking the beginning of the chronic phase of the fasciolosis. This is considered a safe environment for the parasites, away from most of the components of the host innate and acquired immune responses. In this compartment, the liver flukes may live for several decades. They move along the biliary network while feeding on blood, bile, lymph, and tissue fragments, which they use as a source of energy to produce eggs [39]. Although most chronic infections are asymptomatic, pathology at this stage can be severe, and is often related to the number of parasites that reach the bile duct. The physical presence of numerous flukes in the bile duct causes abrasion and even blockage of the bile circulation [244]. Hence, the host species and the host’s overall health, in addition to the parasite species and the burden of infection are all factors that influence pathology during this stage of infection.

The mechanical damage observed during blood feeding within the bile duct is mainly due to the parasites spines, which puncture small blood vessels causing erosion of the epithelium [27,138,245]. In severe infection, extensive damage can cause some parasite eggs to leak out into the liver parenchyma, leading to eosinophilic and granulomatous inflammatory responses [37]. In addition, pathology is exacerbated by the continuous release of fluke molecules into the host biliary network [41,246], which is illustrated by the ability of *F. hepatica* adult ES products alone to induce damage and enlargement of the bile duct [247,248]. As expected, the presence of liver flukes in the bile ducts is marked by a spike of liver enzyme levels, such as GGT and AP, in serum. The increase of AP levels has been linked to the establishment of adult flukes in the bile ducts and hepato-biliary obstruction, which in turn stimulates *de novo* synthesis of the hepatic AP [105]. Indeed, in buffalo infected with *F. gigantica*, bile obstruction was associated with a 107.9% increase in serum AP concentrations [105].

Adult flukes have adapted to survive within the bile. Their tegument is tough and resistant to bile, which consists of a mixture of bile salts, lipids, amino acids, enzymes, and heavy metals, as well as exogenous drugs and toxins that the host consumes [249]. Moreover, the parasites thrive in such an environment by adjusting their metabolism and varying protein expression. For example, the resistance of certain *F. hepatica* isolates to the drug salicylanilide has been linked to increased expression of FhGST [250], and an amino acid substitution in position 143 of the GST was shown to increase TCBZ susceptibility of isolates [251].

The flukes tolerate the low oxygen levels in the bile by expressing high-oxygen affinity hemoglobin [252] and by activating genes involved in anaerobic glycolysis, which allow the fluke to be a facultative anaerobe [253]. Similarly, adult liver flukes can metabolize lipids (i.e., LDL, VLDL, HDL) that are present in large amounts in the bile, and such activity can, eventually, be reflected in the host serum levels of lipids and triglycerides [84,254]. Indeed, humans may develop gallstone disease after months to years of infection, often during the obstructive phase of fasciolosis [142,255,256]. Ultimately, obstruction of the bile ducts arises from both the parasite’s presence and its ES products that cause inflammation and hyperplasia of the epithelium, contributing to the enlargement and mineralization of the bile ducts (cholangitis) and gall bladder (cholecystitis) [255,256].

Similar to the liver-associated stage, the mature adult releases ES products rich in cathepsin L and B peptidases, legumains, peptidase inhibitors, enzymes, glycoproteins and FhHDM (Figure 3) [3,13–15,41,253,257]. Several biochemical and immunological studies have shown the importance of many of these molecules for parasite feeding and detoxification of bile components, as well as their roles in the evasion of host immune responses [170,189,191,210,258–260].

Without doubt, the adult fluke ES products change the bile composition of infected animals but the systemic effects of these molecules released with the bile are still undefined. Through enterohepatic circulation the bile synthesized in the liver is released into the small intestine where it helps in the digestive processes by acting as a detergent. Subsequently, about 95% of the bile contents are reabsorbed in the distal ileum [261]. Hence, the composition of the bile has important implications for the homeostasis of the intestine and liver. During fasciolosis, bile circulation in the gut and liver will transport parasite antigens to distant sites, which may contribute to the immune stimulation observed in the host, even during the chronic phase of the disease [104]. Morphew et al. [262] showed large amounts of *F. hepatica* cathepsin L peptidases in bile fluid collected from naturally infected sheep. Similarly, anti-cathepsin L IgG and IgA antibodies are present in bile, although in considerably lower levels than in serum [263]. These data support the idea that even when hidden in the bile ducts, the adult liver flukes are still capable of stimulating the host immune system. This might explain why the titer of specific anti-cathepsin L antibodies in infected animals’ serum remains high even after the immature stages have left the liver, and only drops after treatment with TCBZ and removal of adult flukes [104,264].

As the main nutrients for adult flukes are derived from blood digestion, at this stage the parasites express several proteins related to host hemoglobin digestion and metabolism, namely FhCL1, leucine aminopeptidases (FhLAP), myoglobin, ferritins, prolylcarboxypeptidase, saposins and FhHDM [12,30,41,259,265]. The process of blood digestion involves the lysis of red blood cells by saposins, releasing hemoglobin that is digested into small peptides by FhCL1, followed by the terminal degradation of hemoglobin peptides by FhLAP (Figure 1) [266–269]. Moreover, heme-binding proteins such as FhHDM play essential roles in detoxifying heme, which is the main product of the metabolism of hemoglobin [270,271]. As *Fasciola* spp. are unable to form hemozoin crystals to eliminate the heme, it secretes high amounts of FhHDM that forms high-molecular weight complexes with heme, inhibiting its harmful peroxidase-associated activity [265,272].

The major components of bile have been investigated by proteomic analysis, which revealed a range of abundant proteins including albumin and immunoglobulins, complement components, coagulation factors (e.g., kallikrein, fibrinogen and anti-thrombin), digestive enzymes such as trypsin, elastase, chymotrypsin and various peptidase inhibitors [14,30,273]. To cope with these host factors, *Fasciola* adult parasites express a range of molecules that are secreted and/or attached to the parasite surface, including serine peptidases inhibitors (serpins). We have recently characterized two *F. hepatica* serpins, FhSrp1 and FhSrp2, which appear to be deliberately expressed to inhibit host chymotrypsin and kallikrein [17]. Both serpins were located on the surface of the immature parasites, but are also highly prevalent in the ES products of all *F. hepatica* life stages [15,17]. The *F. hepatica* serpin family includes seven members, and further characterization of these molecules might link them to the regulation of cascades in which serine peptidases play a central role (e.g., coagulation and complement systems).

Transcriptome and proteome analysis of *F. hepatica* and *F. gigantica* adult parasites and the respective repertoire of secreted proteins from this lifecycle stage has revealed that proteins involved in glycolytic processes are up-regulated [3,183]. Aldolase (fructose-bisphosphate), enolase, and glyceraldehyde 6-phosphate dehydrogenase (GAPDH) are among the enzymes the *Fasciola* spp. flukes either secrete or attach to their tegument surface, where they act mainly as ligands for a variety of host components [3]. As such, these molecules contribute to invasion, modulation of the host’s immune and hemostatic systems, angiogenesis, and acquisition of nutrients [42,274–277]. These enzymes were also characterized as plasminogen-binding proteins, and thus were linked to plasmin generation activity [277]. As plasmin degrades fibrin, this could be the mechanism by which the adult parasites prevent clot formation during blood feeding (Figure 1). In addition, peptides released during fibrin degradation might act as regulators of fibrinogen, reducing fibrin formation that could restrain the parasites.

## *F. gigantica* – not just another fluke

Despite affecting human and livestock health in an area that represents up to 77% of the global population, research interest in *F. gigantica* consistently lags behind that of *F. hepatica* [278]. As a consequence of this neglect, far less is known about the factors contributing to the pathogenicity and virulence of this species. Recent increases in reports of hybrid or introgressed forms between *F. hepatica* and *F. gigantica* in areas where they co-exist suggest the potential for adaptive introgression of various traits between the two species that may enhance their pathogenicity and virulence in mammalian hosts, warranting further investigation [279–282].

The lifecycle of *F. gigantica* follows a similar progression to that of *F. hepatica*, including the reliance on an aquatic intermediate snail host. After infection of the definitive mammalian hosts by ingestion, *F. gigantica* metacercariae excyst in the small intestines before reaching the liver via the abdominal cavity, where they migrate for a period of up to 16 weeks [283–285]. Mature adults reside in the bile ducts of infected hosts and shed eggs into their feces. Under optimal conditions, the eggs of *F. gigantica* hatch after a period of 10–11 days, releasing the short-lived miracidia that must find a suitable intermediate snail hosts to continue the parasite lifecycle [286].

The lifecycle similarities between *F. hepatica* and *F. gigantica* have led to the erroneous assumption that their epidemiologies, and therefore their ability to cause disease in infected mammalian hosts, are similar. Differences between the susceptibility of various species and breeds of mammalian hosts, on the other hand, have prompted the conclusion that *F. gigantica* is less virulent than *F. hepatica*. As it stands, however, there is limited empirical data available to support current conclusions comparing the pathogenicity and virulence of *F. gigantica* to that of *F. hepatica*, and existing information must be interpreted with an appreciation for the differences between these two parasites.

### Virulence and pathogenicity in mammalian hosts

Several studies have attempted to compare the virulence of *F. gigantica* to *F. hepatica* in mammalian hosts by contrasting the percentage take of infective metacercariae during experimental infections along with the impact of infection on immunological and biochemical markers [287–294]. A reliance on small sample sizes (1–5 animals/group) and large varying infectious doses (500–20,000 metacercariae/dose) have limited the statistical significance of these findings and their application to our understanding of natural infections, making it difficult to determine if one species is truly more virulent and/or pathogenic than the other. The length of time post infection has also been shown to influence the number of parasites available for recovery from the liver, with fewer *F. gigantica* adults present in the livers of infected cattle from 5 months post infection [284]. What is clear, however, is that a difference in susceptibility to *F. gigantica* infection exists not only between different species of mammalian hosts, but also between different breeds within the same host species. These differences may help us to infer the mechanisms of innate and acquired resistance and immune-based pathogenesis against infection with *F. hepatica* and *F. gigantica*, as well as help us shed light on the defense mechanisms employed by the parasites when under attack by the host’s immune response.

Swamp buffalo appear to be the most resilient mammalian host to infection with *F. gigantica*, as demonstrated by lower parasite burdens, reduced fecal egg counts, less apparent clinical signs and less significant impact on biochemical parameters such as packed cell volume (PCV), GGT and LDH compared to various *Bos indicus* cattle breeds exposed to the same infectious dose [285,295–297]. Global serum, liver, hepatic lymph node and spleen proteome analysis has recently been conducted on experimentally infected riverine buffaloes in order to elucidate the mechanisms of host responses to infection during the invasive (3–10 days post infection; DPI), early (28–70 DPI) and late (≥ 98 DPI) stages of infection [298]. These analyses have revealed the downregulation of metabolic processes in infected host liver throughout infection and a shift toward redox processes during early infection, likely as a form of offense against the invading immature flukes [298]. Similarly, Indonesian thin tail (ITT) sheep have been shown to have a high level of resistance to infection against *F. gigantica* via the generation of both a strong innate and adaptive immune response [287–289,291]. Interestingly, ITT are susceptible to infection with *F. hepatica*, suggesting that this parasite species is more adept at modulating the host response to infection [287,291]. Proteomic and transcriptional analyses of liver, serum and hepatic lymph nodes during experimental infection with *F. hepatica* and *F. gigantica* in this species and its comparison to existing datasets from water buffalo may help shed light on the exact processes involved in parasite invasion and evasion of host immune responses.

Recent studies by Zhang et al. [299] applied proteomic techniques to identify a signature of *F. gigantica* infection in serum from swamp buffaloes at 3, 42 and 70 DPI. Six significantly up-regulated proteins were identified in infected serum compared to uninfected buffaloes, namely MHC I antigen, microglobulin, NID2 protein, fetuin-B and fibrinogen gamma-B chain. Histopathological examination of hosts infected with *F. gigantica* revealed cellular infiltration, hemorrhage and fibrosis without calcification in the liver parenchyma, which increased over the course of infection [300]. This pathogenesis has been attributed to the suppression of the host’s pro-inflammatory responses, emphasized by low levels of cytokines such as interleukin-1β (IL-1β), IL-2, IL-6, IL-12, and IFN-γ [301], and changing in the expression profile of genes involved in TLRs and NOD-like receptors (NLRs) signaling pathways in serum, liver and peripheral blood mononuclear cell (PBMC) of infected buffaloes [302]. During the early stages of infections (3–13 DPI), a mixed Th1- and Th2-type immune response is observed, which is thought to facilitate the parasite’s establishment [300,301,303]. Conversely, systemic immunological analysis of the serum and lymphoid organs of infected animals 98 DPI revealed that during chronic infection the host responses are completely skewed toward a Th2 pattern. This is illustrated by enhanced expression of IL-4 and the IgG1 antibody isotype [300,303]. Furthermore, the strength of the Th2 response elicited is thought to be indicative of the susceptibility of the host species to *F. gigantica* infection [303]. Unlike *F. hepatica*, factors responsible for modulating the immune response during infection with *F. gigantica* remain largely unknown. However, studies have demonstrated that *F. gigantica* ES products play an important role by suppressing maturation of immune cells such as DCs [304], as well as altering the expression of genes associated with the host immune responses, receptor signaling, disease and metabolism [305]. Recent mass spectrometry analysis of *F. gigantica* ES has identified many of the same virulence associated proteins involved in immune modulation by *F. hepatica*, including cathepsin L and B peptidases, antioxidants and FABPs [306], but as to whether they exert similar modulatory effects remains to be determined.

There are fewer reports of human infections with *F. gigantica* than with *F. hepatica*, leading to the assumption that *F. hepatica* is more pathogenic in areas where human fasciolosis is common [46,255,307]. The tendency for *F. gigantica* to occur in less-developed regions where access to medical facilities is limited, however, suggests that perhaps human cases of *F. gigantica* infection are simply underreported. There are also suggestions that *F. gigantica* may be less virulent in human infections, resulting in a milder form of disease that causes less pain and therefore goes unnoticed for longer [307,308]. The production of a higher proportion of floating cysts by *F. gigantica* compared to *F. hepatica* may provide additional sources of infection such as via the use of contaminated water for washing otherwise safe vegetables or through drinking [76]. The occurrence of *F. gigantica* in regions maintaining the livelihoods of up to 6 billion people further supports the suggestion that human cases of *F. gigantica* infection are equally – if not more – prevalent than *F. hepatica*, and are simply undocumented.

## Fasciola*-hybrid or introgressed forms*

Increasing reports of hybridization and/or introgression between *F. hepatica* and *F. gigantica* have raised the possibility of the existence of *Fasciola* spp. with intermediate pathogenicity and virulence traits [278]. Experimentally, hybridization between *F. hepatica* and *F. gigantica* has been demonstrated under laboratory conditions and the continued identification of these forms in field samples suggests that they are a continually occurring phenomenon [309–311]. While studies on the functional implications of these genetic events are currently unavailable, lab-maintained *Fasciola*-hybrid adults demonstrated an intermediate body size between that of their parent species and are considered more infectious in Wistar rats than *F. gigantica* alone based on higher recovery rates [309]. Hybridization between these two parasites may not necessarily generate permanent hybrid strains and yet the potential for introgression of advantageous traits between these two species as a result of the back-crossing of hybrids is worthy of further consideration [278].

Increasing areas of parasite sympatry as a result of international livestock movements, combined with climate change-derived shifts in conditions suitable for the survival of both species, suggests that future work should be directed toward understanding the potential human and animal health risks associated with these genetic events, including potential impacts on their pathogenicity and virulence [106,278]. Furthermore, as TCBZ resistant *F. hepatica* continue to spread across the globe the potential for the emergence of drug-resistant *Fasciola* hybrids should be closely monitored, particularly since this is the only drug available for the treatment of human fasciolosis.

## Conclusion

Fasciolosis caused by flatworms of the species *Fasciola* have been scourges of farmed animals for centuries but only in recent decades has their zoonotic importance become realized. There is no vaccine available and, despite much progress in understanding the biology of the parasites and experimental research toward this goal, it is unlikely that we will see one in the next five years. Meanwhile, parasites that are resistant to frontline drugs such as triclabendazole are continuing to spread globally leaving farmers and veterinarians without a means of controlling on-farm disease and medics without an effective treatment for human infection with drug-resistant parasites. Climate change is also impacting on the prevalence and distribution of the disease [120] and live animal trade is helping to fast-forward the spread of new species or isolates to new regions as well has promoting the expansion of hybrid *F. hepatica/gigantica* parasite forms [278]. Research advances are therefore indispensable to overcome these issues. As shown in this review, great advances in molecular biology, genomics/genetics and -omics are allowing us to develop a detailed molecular picture of parasite infection, virulence and pathogenicity. This information is advancing our understanding of the parasite-host interactions, enabling the development of effective control strategies (vaccines and drugs), as well as diagnostic tools that will ultimately allow us to identify and treat infections, which is fundamental to prevent both disease spread and economic losses that result from both *F. hepatica* and *F. gigantica*.

## Data Availability

Data sharing is not applicable to this article as no new data were created or analysed in this study.
